# Assessment of salivary gland function after head and neck radiotherapy using 3DCRT and VMAT: a comparative clinical study

**DOI:** 10.3389/froh.2026.1934444

**Published:** 2026-10-14

**Authors:** Sonja Rogoleva Gjurovski, Mihajlo Petrovski, Lenche Kostadinova, Ljupka Arsovski, Verica Toneva Stojmenova, Katerina Zlatanovska, Sandra Atanasova, Ljubica Prosheva Pelivanova, Bruno Nikolovski

**Affiliations:** Faculty of Medical Sciences, Goce Delcev University, Stip, North Macedonia

**Keywords:** hyposalivation, malignant neoplasms, radiation-induced complications, radiotherapy, salivary flow, salivary hypofunction, xerostomia

## Abstract

**Introduction:**

Salivary gland hypofunction is a common consequence of radiotherapy in patients with head and neck malignancies. Reduced salivary secretion may result in hyposalivation and may contribute to oral dryness and other oral complications. This study compared objective measures of salivary gland function and oral mucosal moisture in patients treated with three-dimensional conformal radiotherapy (3DCRT) and volumetric modulated arc therapy (VMAT) three months after completion of radiotherapy.

**Material and methods:**

This comparative cross-sectional study included 60 patients with confirmed oropharyngeal malignancies evaluated three months after completion of radiotherapy. Thirty patients had been treated with three-dimensional conformal radiotherapy (3DCRT), while 30 patients with oropharyngeal malignancies had been treated with volumetric modulated arc therapy (VMAT) combined with concurrent weekly cisplatin. Unstimulated whole saliva was collected using the spitting method into SimplOFy collection tubes. Oral mucosal moisture was assessed using a modified Schirmer test. Following a 30-minute interval, stimulated whole saliva was collected using SimplOFy tubes after electrical stimulation with a SaliPen device. Stimulated parotid salivary flow was subsequently assessed using modified Lashley cups with gustatory stimulation.

**Results:**

All four evaluated parameters showed statistically significant differences between the treatment groups. Median unstimulated salivary flow was 0.07 (IQR 0.05–0.08) mL/min in the 3DCRT group and 0.14 (IQR 0.10–0.20) mL/min in the VMAT group (*p* < 0.001). Median stimulated whole-saliva flow was 0.25 (IQR 0.20–0.30) mL/min and 0.50 (IQR 0.40–0.60) mL/min, respectively (*p* < 0.001). Median stimulated parotid salivary flow was 0.10 (IQR 0.09–0.12) mL/min in the 3DCRT group compared with 0.24 (IQR 0.20–0.29) mL/min in the VMAT group (*p* < 0.001). Median oral mucosal moisture was 7 (IQR 5–8) mm in the 3DCRT group and 13 (IQR 11–18) mm in the VMAT group (*p* < 0.001).

**Conclusion:**

Three months after radiotherapy, patients treated with VMAT demonstrated significantly higher unstimulated and stimulated salivary flow rates, stimulated parotid salivary flow, and oral mucosal moisture than patients treated with 3DCRT. These findings suggest better objective salivary function in the VMAT cohort; however, the cross-sectional post-treatment design and potential differences in clinical and treatment-related characteristics between the groups preclude conclusions regarding causality or definitive preservation of salivary gland function.

## 1 Introduction

Salivary gland hypofunction is a common consequence of radiotherapy in patients with malignant head and neck neoplasms, resulting in significant reductions in salivary flow and pronounced hyposalivation, which may subsequently manifest as the subjective sensation of xerostomia. The extent of salivary impairment varies according to the radiotherapy technique, radiation dose and intensity, and treatment schedule. These factors may also influence the severity of post-radiation oral complications and contribute to alterations in the biochemical composition of saliva.

Because of the high radiosensitivity of the salivary glands, salivary flow is most often reduced by approximately 50%–60% within the first week of radiotherapy. This also affects saliva composition, which is reflected in changes in its viscosity, electrolyte levels, buffering capacity, and pH value. In addition, alterations occur in salivary mucoglycoproteins, which are responsible for its viscoelastic properties, lubrication, and adhesion to the oral epithelium (1).

Despite all efforts to spare surrounding healthy tissues during radiotherapy, the salivary glands are inevitably exposed to radiation due to their anatomical proximity, leading to unavoidable damage and subsequent consequences. The severity of these effects is mainly associated with the total radiation dose, dose per fraction, irradiated volume, dose distribution within the tissue volume, combination with chemotherapy, and overall treatment duration (2). The exact mechanism by which radiotherapy affects the salivary glands is still not fully understood, although the major salivary glands are exposed to relatively high and significant radiation doses. Histological findings commonly reveal changes such as acinar atrophy and degeneration (2, 3).

Three-dimensional conformal radiotherapy (3DCRT) and intensity-modulated radiotherapy (IMRT) are widely used techniques for the treatment of head and neck malignancies, with important implications for the preservation of salivary gland function. While 3DCRT is a well-established conventional technique, IMRT enables more precise dose distribution through modulation of radiation beam intensity, with the aim of limiting radiation exposure to surrounding healthy tissues. Volumetric modulated arc therapy (VMAT), an advanced form of IMRT, further optimizes treatment delivery by continuously rotating the gantry while simultaneously modulating beam intensity and dose rate around the target volume. From a dental and oral health perspective, preservation of salivary gland function is particularly important, as reduced salivary secretion may contribute to hyposalivation, xerostomia, and subsequent oral complications. Recent studies have suggested that VMAT may provide better protection of salivary glands, resulting in improved salivary function and a lower incidence of xerostomia compared with conventional radiotherapy techniques (4–7).

The aim of this study was to compare objective measures of salivary gland function, including unstimulated and stimulated whole-saliva flow, stimulated parotid salivary flow, and oral mucosal moisture, in patients assessed three months after treatment with 3DCRT or VMAT.

## 2 Material and method

Salivary flow measurements were performed at the University Clinic of Maxillofacial Surgery following completion of radiotherapy. Patients were selected at the University Clinic for Radiotherapy and Oncology—Skopje according to predefined inclusion and exclusion criteria. All participants provided written informed consent prior to inclusion in the study.

The study included 60 patients with histologically confirmed oropharyngeal malignancies who fulfilled the predefined inclusion and exclusion criteria during the study period. Тhe inclusion criteria were: age ≥18 years; both sexes; and histologically diagnosed oropharyngeal malignancy treated with radiotherapy using either VMAT or 3D conformal radiotherapy (3DCRT), irrespective of whether the patient had undergone surgery or was considered inoperable. The exclusion criteria were: salivary gland dysfunction attributable to causes other than radiotherapy; autoimmune diseases, including systemic lupus erythematosus; Sjögren syndrome; metabolic disorders; and sialolithiasis or sialadenitis.

Patients were categorized into two groups according to the radiotherapy technique they received: 30 patients were treated with conventional three-dimensional conformal radiotherapy (3DCRT), while 30 patients were treated with volumetric modulated arc therapy (VMAT). The allocation to 3DCRT or VMAT was determined individually by the radiation oncologist based on the radiotherapy plan developed by the medical physicists following tumor delineation and treatment planning, with the radiotherapy modality considered most appropriate and acceptable for the individual patient being selected. The allocation was not randomized. All patients in both groups received concurrent weekly chemotherapy with cisplatin according to the institutional treatment protocol. Thus, both groups included patients with oropharyngeal malignancies and received concurrent weekly cisplatin, while the principal treatment difference between the groups was the radiotherapy technique.

An *a priori* sample size calculation was not performed, as the study included all available eligible patients during the defined study period. Patient selection and enrollment were performed three months after completion of radiotherapy in order to minimize variability related to the timing of post-radiotherapy salivary function assessment.

Patients in both groups underwent radiotherapy according to standard oncological treatment protocols. In the VMAT group, after immobilization with a thermoplastic mask and CT simulation, treatment was delivered using a Halcyon system according to an individualized VMAT treatment plan. A total dose of 60 Gy in 30 fractions (2 Gy per fraction) was prescribed to the primary tumor planning target volume (PTV60), with dose escalation to 66 or 70 Gy according to individual tumor characteristics and treatment requirements. Patients in the 3DCRT group were treated using a linear accelerator with an individualized three-dimensional conformal treatment plan. The total prescribed dose ranged from 60 to 70 Gy, depending on tumor characteristics, surgical status, and pathological risk factors. In both groups, elective irradiation of the regional lymphatic areas was performed to a total dose of 50 Gy according to DAHANCA guidelines.

### 2.1 Assessment of salivary flow

Salivary function was assessed three months after completion of radiotherapy according to a standardized protocol. Before each measurement, participants were instructed not to eat, smoke, or use oral hygiene products for at least 30 min prior to sampling.

Unstimulated whole saliva flow was measured using the spitting method. Saliva was collected into specialized SimplOFy collection devices (Figure 1), which have a maximum capacity of 2 mL and are designed as graduated collection tubes with a funnel to facilitate saliva collection, stabilization, and storage.

**Figure 1:**
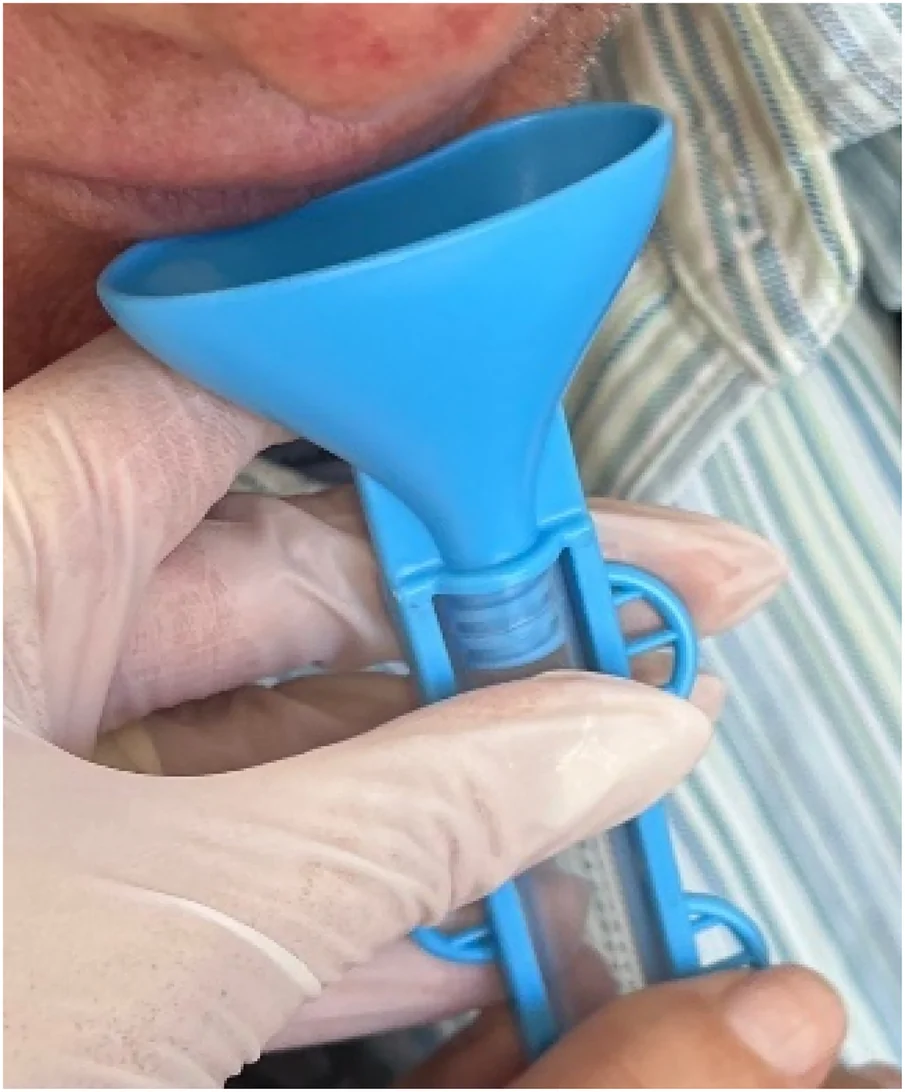
Saliva collection in graduated test tubes.

Immediately before collection, participants rinsed their mouths with water and then waited for 5 min. During the collection period, participants allowed saliva to accumulate in the mouth and subsequently expectorated it into the SimplOFy collection tube. The collected volume was recorded and expressed as mL/min.

Oral mucosal moisture was assessed using a modified Schirmer test. Standardized Schirmer strips were placed in contact with the floor of the oral cavity. The strips remained in position for 3 min, after which the length of the moistened portion was measured in millimeters. Measurements were recorded on a scale ranging from 0 to 35 mm, with higher values indicating greater oral mucosal moisture (Figure 2).

**Figure 2:**
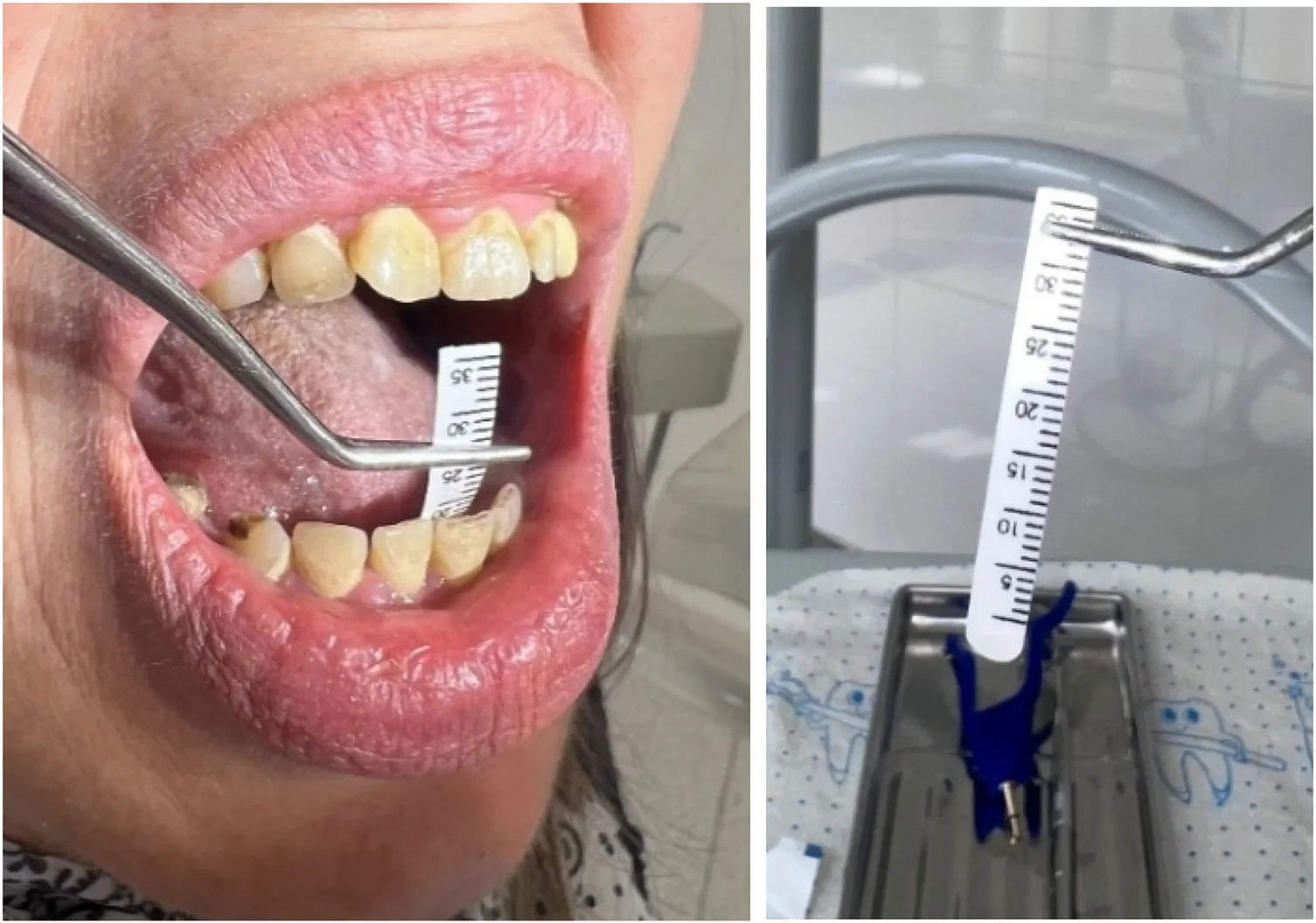
Modified method for measuring oral moisture.

Following a 30-minute interval after the unstimulated saliva measurement, stimulated whole saliva was collected using SimplOFy collection tubes. Salivary secretion was stimulated using a SaliPen (Figure 3) electrical stimulation device according to the standardized measurement protocol. These devices generate repetitive electrical impulses that stimulate the nerves responsible for salivary secretion, thereby increasing salivary gland activity and secretion. The device includes an intraoral attachment with two electrodes placed on the lingual side and at the most distal part of the alveolar process. The volume of stimulated whole saliva collected during the defined collection period was recorded and expressed as mL/min (Figure 4).

**Figure 3:**
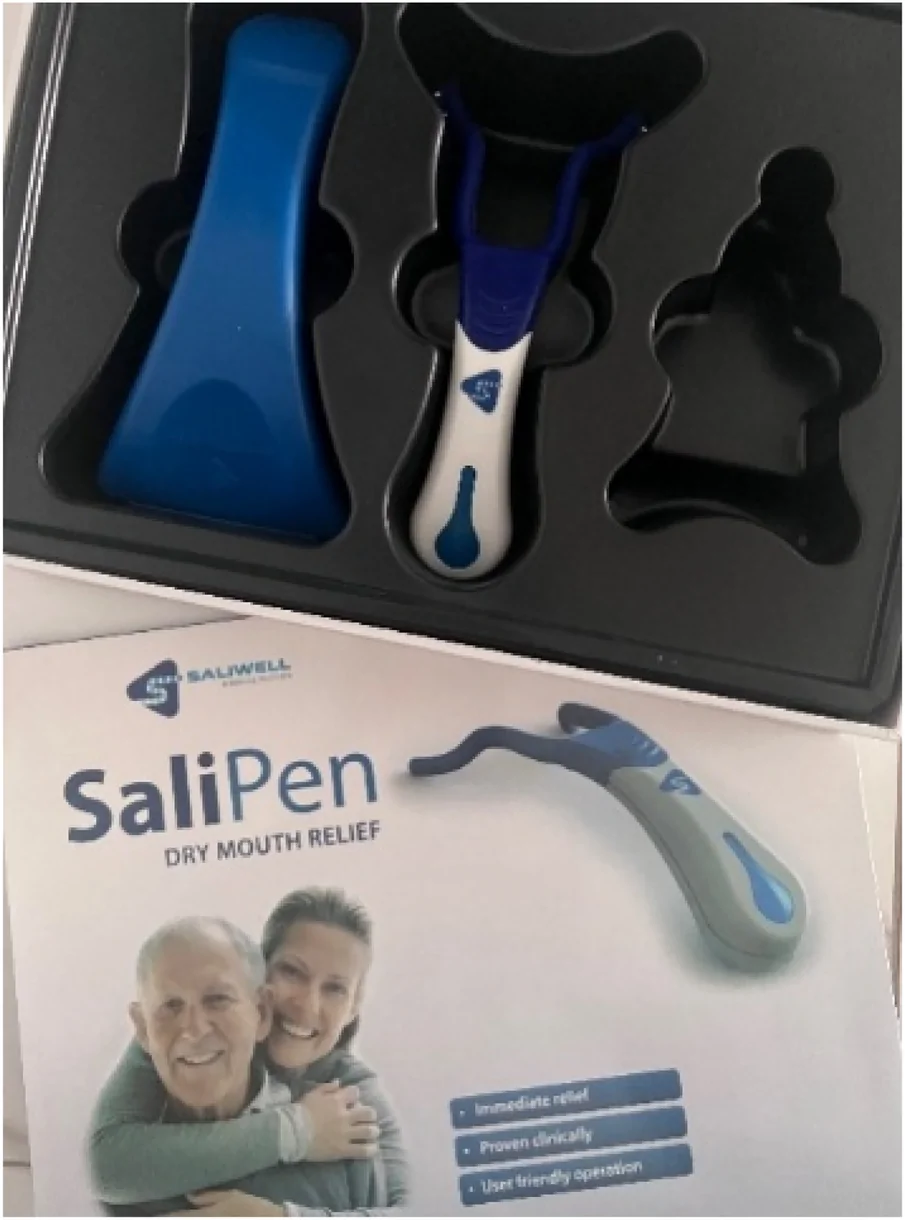
Salipen—electrical stimulation device.

**Figure 4:**
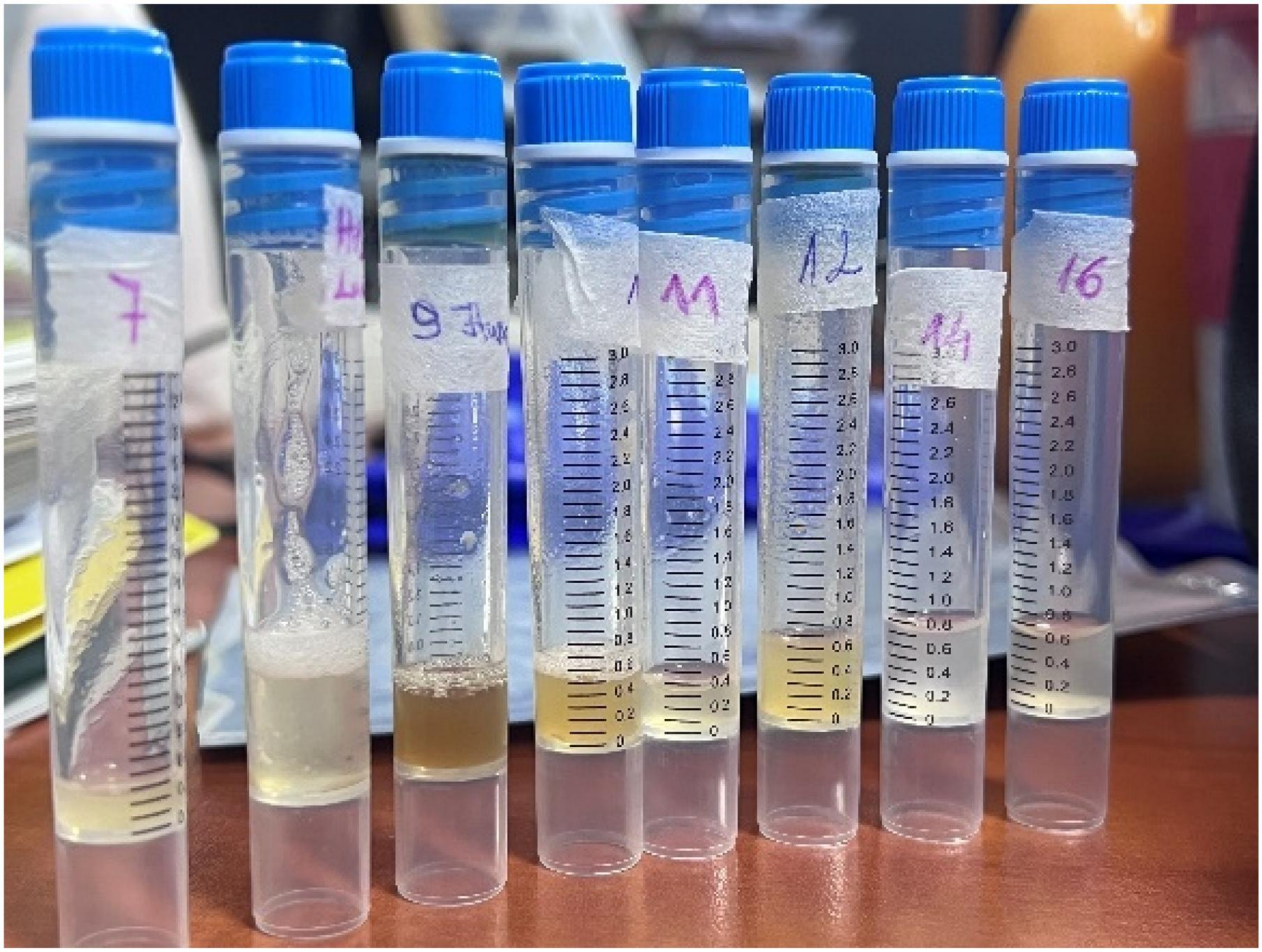
Test tubes with collected saliva.

Stimulated parotid saliva was collected separately using devices based on the principle of modified Lashley cups (Figure 5), fabricated in a dental laboratory from silicone impression material and connected to a tubing system allowing simultaneous collection from both parotid glands. The collection device was positioned over the opening of the parotid duct, allowing saliva secreted from the parotid gland to be collected separately from the oral cavit. Prior to collection, salivary secretion from the parotid gland was stimulated using 2% citric acid applied with a micropipette to the dorsal and dorsolateral surfaces of the tongue five times over 2 min, with total collection lasting 30 min. The final volume was divided by 30 to obtain mL/min. In patients where measurements could not be obtained, sampling was rescheduled for the following day.

**Figure 5:**
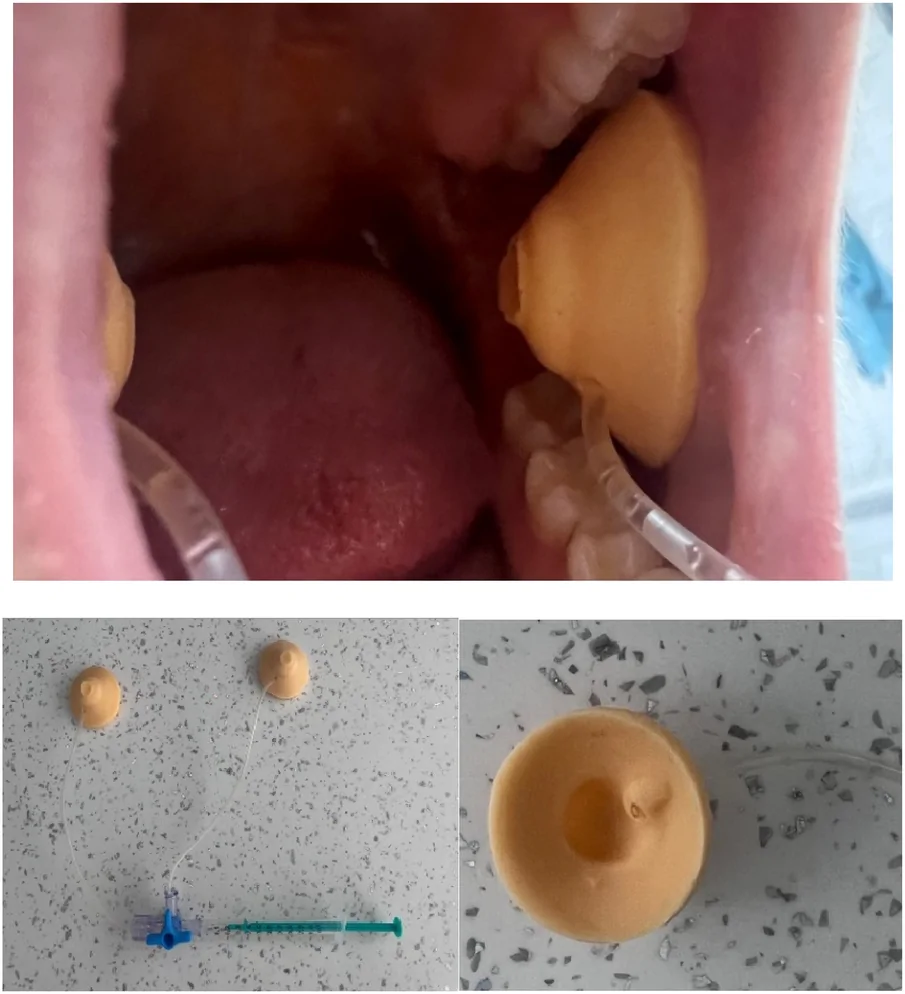
Modified lashley cups.

Stimulated whole saliva was collected using SimplOFy tubes following electrical stimulation with a SaliPen device. The SaliPen generates repetitive electrical impulses that stimulate the neural pathways involved in salivary secretion, thereby increasing overall salivary gland activity. The intraoral attachment contains two electrodes positioned on the lingual side and at the most distal part of the alveolar process. In contrast, stimulated parotid saliva was collected selectively using modified Lashley cups positioned over the openings of the Stensen's ducts, with salivary secretion stimulated by application of 2% citric acid to the dorsal and dorsolateral surfaces of the tongue. Thus, electrical stimulation was used to assess overall stimulated whole-saliva secretion, whereas gustatory stimulation with citric acid combined with selective ductal collection was used to specifically assess parotid gland secretion. The two stimulation methods were selected according to the different measurement objectives: assessment of total stimulated salivary flow versus specific evaluation of parotid gland secretion.

All salivary flow and oral mucosal moisture measurements were performed according to the same standardized protocol and by trained personnel in order to minimize methodological variability between the two treatment groups.

The data collected from the study participants were statistically analyzed using the SPSS software package (version 26.0 for Windows; SPSS Inc., Chicago, IL, USA).

The study was approved by the Ethics Committee (Approval No. 02-1511/2).

## 3 Results

The study included 60 patients, of whom 37 (61.7%) were male and 23 (38.3%) were female, with a mean age of 57.33 ± 9.83 years (range, 26–72). The 3DCRT group included 22 males and 8 females, while the VMAT group included 15 males and 15 females (Table 1).

**Table 1:** Baseline characteristics of patients according to radiotherapy technique.

Baseline characteristic	3DCRT (*N* = 30)	VMAT (*N* = 30)	Total (*N* = 60)	*p*-value
Age, years, mean ± SD	62.57 ± 7.21	52.10 ± 9.37	57.33 ± 9.83	<0.001
Age range, years	50–72	26–65	26–72	
Sex, *n* (%)				
Male	22 (73.3%)	15 (50.0%)	37 (61.7%)	
Female	8 (26.7%)	15 (50.0%)	23 (38.3%)	
Previous surgery, *n* (%)				
Yes	25 (83.3%)	24 (80.0%)	49 (81.7%)	
No	5 (16.7%)	6 (20.0%)	11 (18.3%)	
Primary tumor site	Oropharyngeal	Oropharyngeal	Oropharyngeal	
Concurrent chemotherapy	Weekly cisplatin	Weekly cisplatin	Weekly cisplatin	

Age was compared between groups using the Mann–Whitney U test.

Within the study, salivary gland function was assessed in the patients by analyzing collected saliva samples with respect to the following parameters: (1) unstimulated salivary flow rate (mL/min); (2) stimulated salivary flow rate (mL/min); (3) stimulated parotid salivary flow rate (mL/min); and (4) oral mucosal moisture measured using the Schirmer test (mm). These parameters were analyzed for the entire study sample comprising 60 (100%) patients. In addition, a separate analysis and comparison were performed between the two study groups (3DCRT and VMAT) (Table 2; Figures 7–10).

**Table 2:** Comparison of the 3DCRT and VMAT groups based on selected quantitative parameters of salivary gland function.

Parameters	N	Mean ± SD	Min/Max	25th	50th (Median)	75th	p^1^
Unstimulated salivary flow rate (mL/min)							
** **3DCRT	30	0.06 ± 0.02	0.0/0.1	0.05	0.07	0.08	Z = −6.461; *p* **<** 0.001*
** **VMAT	30	0.16 ± 0.08	0.1/0.4	0.10	0.14	0.20	
Total	60	0.11 ± 0.08	0.0/0.4	0.07	0.09	0.14	
Stimulated salivary flow rate (mL/min)							
** **3DCRT	30	0.26 ± 0.08	0.2/0.5	0.20	0.25	0.30	Z = −6.276; ***p*** **<** 0.001*
** **VMAT	30	0.56 ± 0.18	0.4/1.2	0.40	0.50	0.60	
Total	60	0.41 ± 0.20	0.1/1.2	0.25	0.40	0.50	
Stimulated parotid salivary flow rate (mL/min)							
** **3DCRT	30	0.11 ± 0.04	0.0/0.2	0.09	0.10	0.12	Z = −6.490; ***p*** **<** 0.001*
** **VMAT	30	0.25 ± 0.07	0.1/0.5	0.20	0.24	0.29	
Total	60	0.17 ± 0.10	0.0/0.5	0.10	0.17	0.24	
Oral mucosal moisture (mm)							
** **3DCRT	30	7.00 ± 3.84	2/25	5	7	8	Z = −6.246;***p*** **<** 0.001*
** **VMAT	30	15.43 ± 6.34	9/33	11	13	18	
Total	60	11.21 ± 6.71	2/33	7	10	13	

3DCRT, Three-Dimensional Conformal Radiotherapy; VMAT, Volumetric Modulated Arc Therapy; Mean, Mean; SD, Standard Deviation; Median, Median; Min/Max, Minimum/Maximum; Percentiles, Percentiles.

Z=Mann–Whitney U test.

*Significant at *p* **<** **0.05**.

The analysis of the obtained values for the selected quantitative parameters of salivary gland function indicated a non-normal distribution of the data. The Shapiro–Wilk test demonstrated statistically significant deviations from normality for all four parameters: unstimulated saliva (W = 0.8031, *p* < 0.001), stimulated saliva (W = 0.8902, *p* < 0.001), stimulated saliva from the parotid gland (W = 0.9540, *p* = 0.024), and oral mucosal moisture (W = 0.8612, *p* < 0.001) (Figure 6). Based on these findings, non-parametric statistical tests were used for subsequent comparisons between the 3DCRT and VMAT groups.

**Figure 6:**
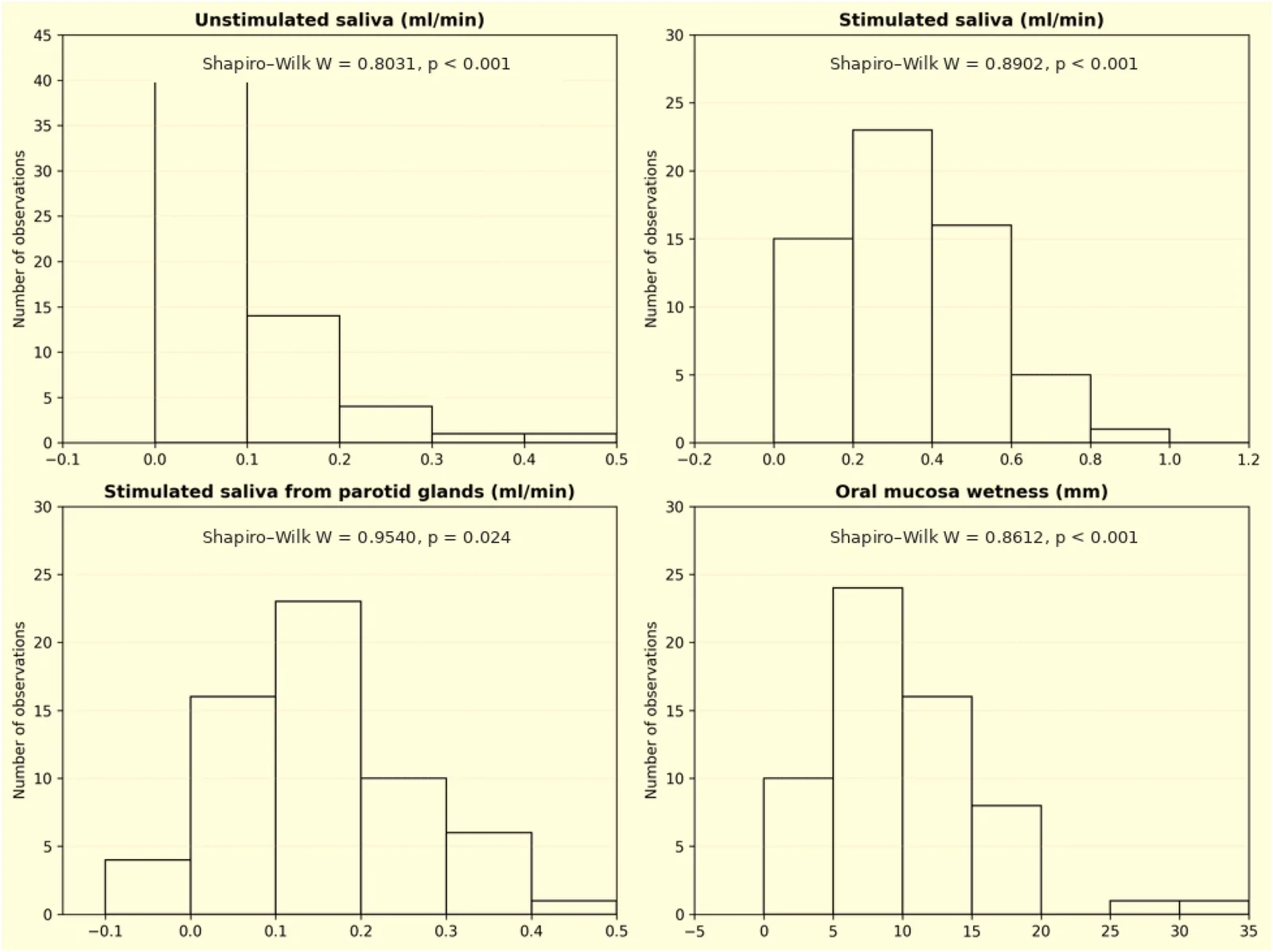
Analysis of selected parameters for salivary gland function.

### 3.1 Unstimulated salivary secretion (mL/min)

The unstimulated salivary flow rate differed significantly between the two radiotherapy groups. Patients treated with VMAT demonstrated higher unstimulated salivary flow compared with those treated with 3DCRT. The median (IQR) salivary flow rate was 0.14 (0.10–0.20) mL/min in the VMAT group, compared with 0.07 (0.05–0.08) mL/min in the 3DCRT group. The between-group difference was statistically significant according to the Mann–Whitney U test (Z = −6.461; *p* < 0.001), indicating substantially higher unstimulated salivary output among patients treated with VMAT (Table 2; Figure 7).

**Figure 7:**
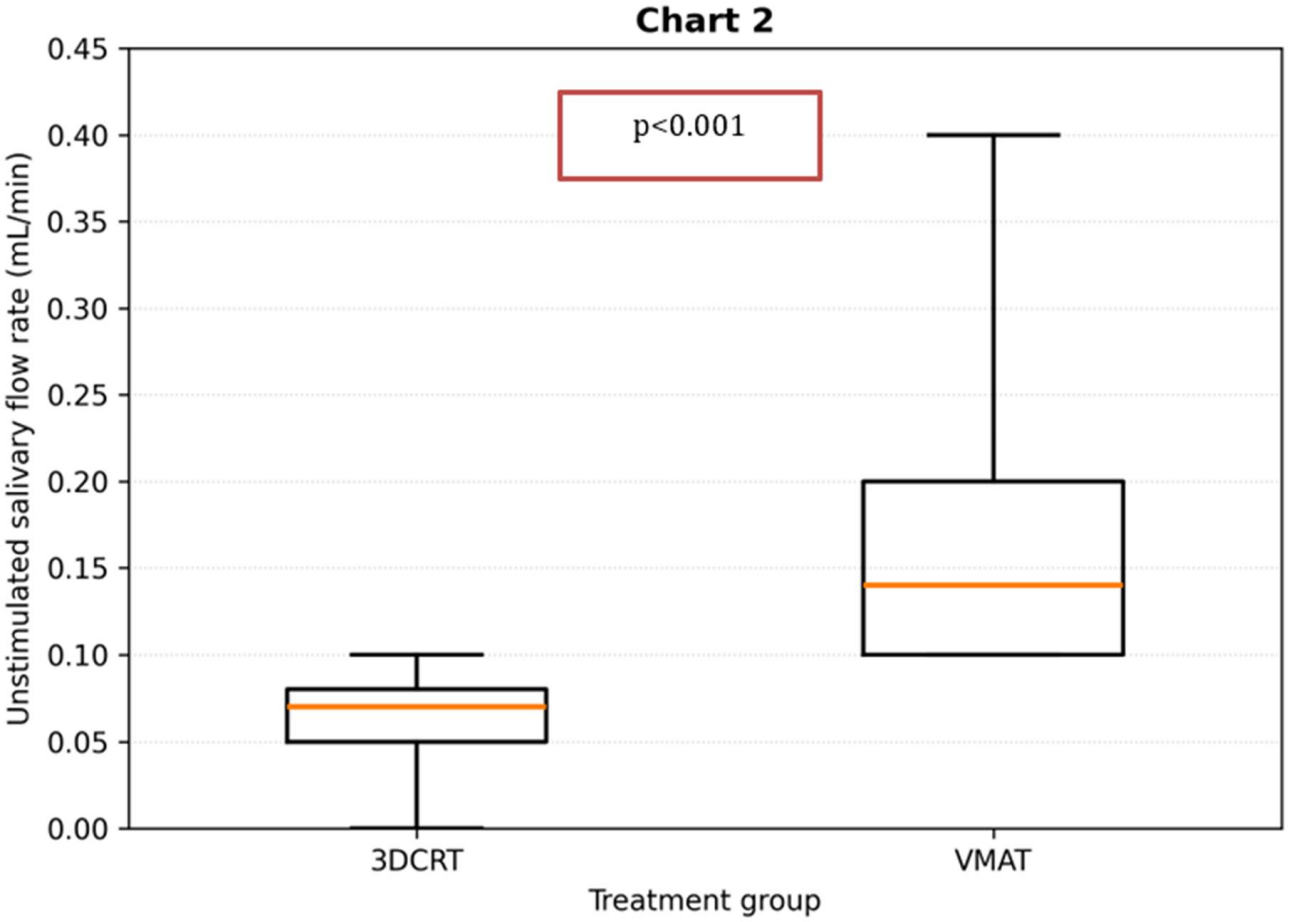
Analysis by groups (3DCRT/VMAT) and unstimulated saliva.

### 3.2 Stimulated salivary secretion (mL/min)

A statistically significant difference was also observed in stimulated whole salivary flow between the two groups. The VMAT group demonstrated higher stimulated whole salivary flow than the 3DCRT group, with a median (IQR) value of 0.50 (0.40–0.60) mL/min compared with 0.25 (0.20–0.30) mL/min, respectively. The Mann–Whitney U test confirmed a statistically significant between-group difference (Z = −6.276; *p* < 0.001). These findings indicate a higher stimulated whole salivary output in patients treated with VMAT compared with those treated with 3DCRT (Table 2; Figure 8).

**Figure 8:**
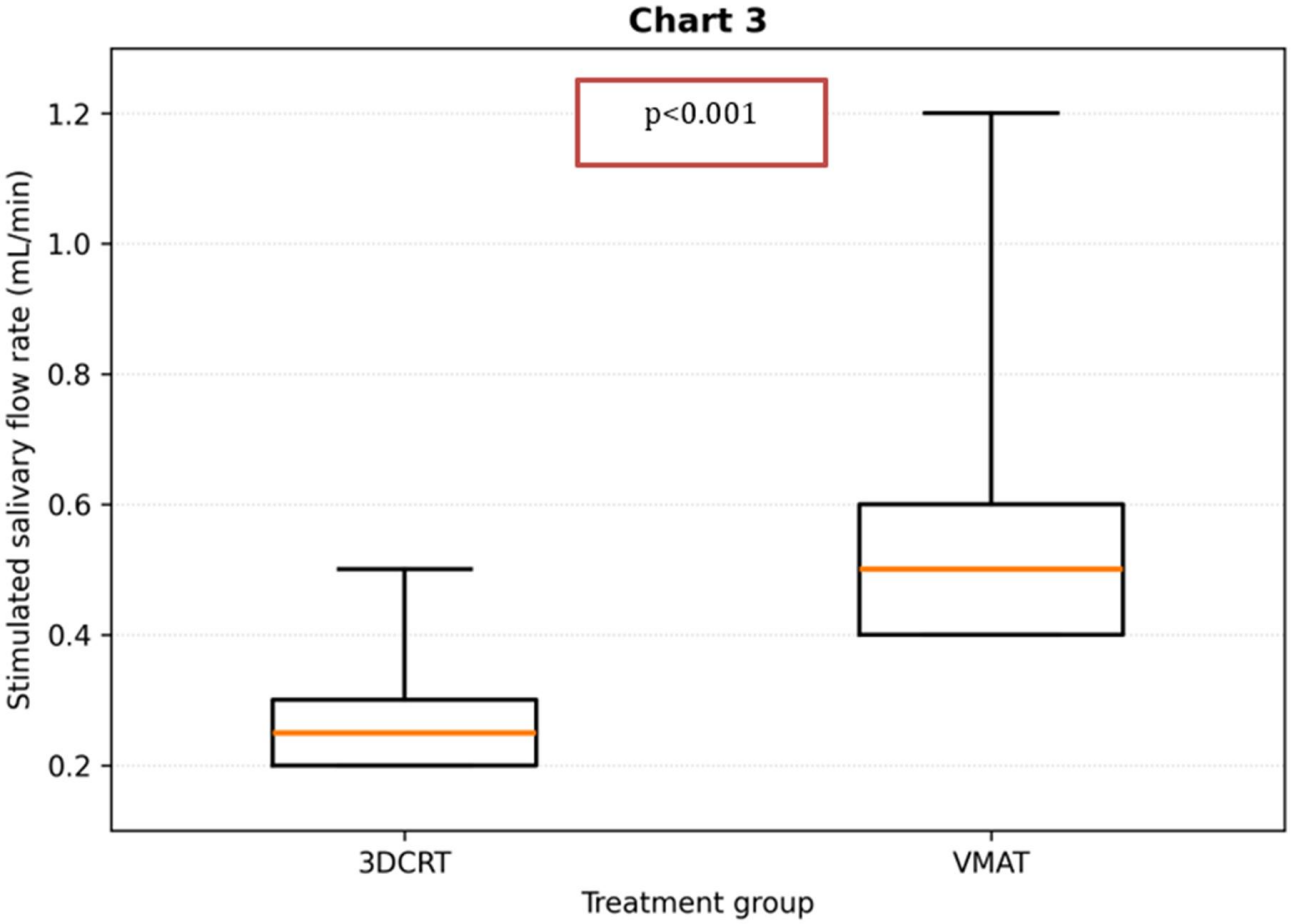
Analysis by groups (3DCRT/VMAT) and stimulated saliva.

### 3.3 Stimulated parotid salivary secretion (mL/min)

The stimulated parotid salivary flow rate was also significantly higher in the VMAT group than in the 3DCRT group. The median (IQR) stimulated parotid flow was 0.24 (0.20–0.29) mL/min in patients treated with VMAT, compared with 0.10 (0.09–0.12) mL/min in the 3DCRT group. The observed difference between the groups was statistically significant according to the Mann–Whitney U test (Z = −6.490; *p* < 0.001), demonstrating higher stimulated parotid salivary output in the VMAT cohort (Table 2; Figure 9).

**Figure 9:**
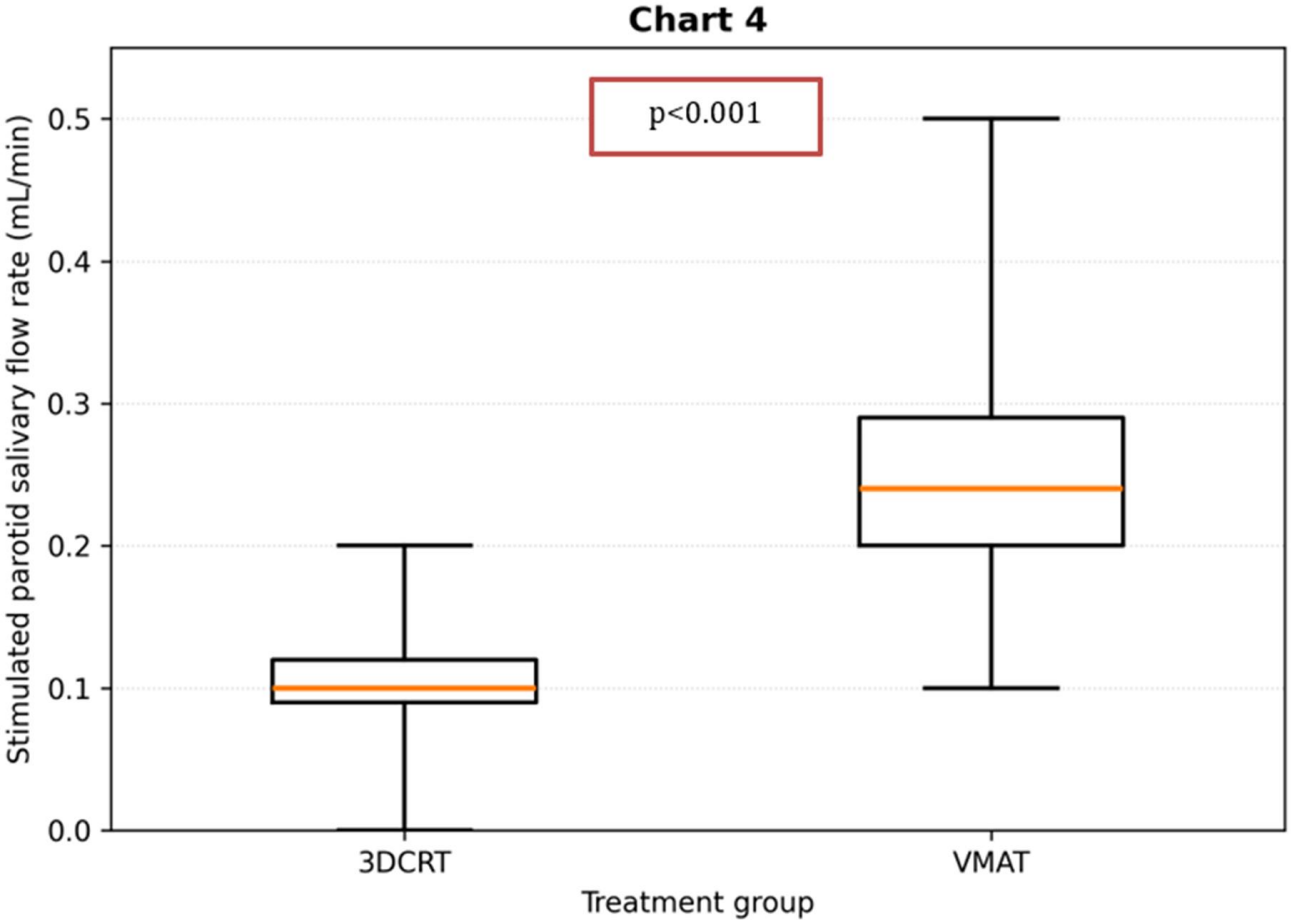
Analysis according to groups (3DCRT/VMAT) and stimulated salivary secretion from the parotid gland.

### 3.4 Oral mucosal moisture (mm)

A statistically significant difference was observed in oral moisture between the two radiotherapy groups. Patients in the VMAT group showed higher oral moisture measurements than those in the 3DCRT group. The median (IQR) oral moisture value was 13 (11–18) mm in the VMAT group, compared with 7 (5–8) mm in the 3DCRT group. The Mann–Whitney U test demonstrated a statistically significant difference between the groups (Z = −6.246; *p* < 0.001), indicating higher measured oral moisture among patients treated with VMAT (Table 2; Figure 10).

**Figure 10:**
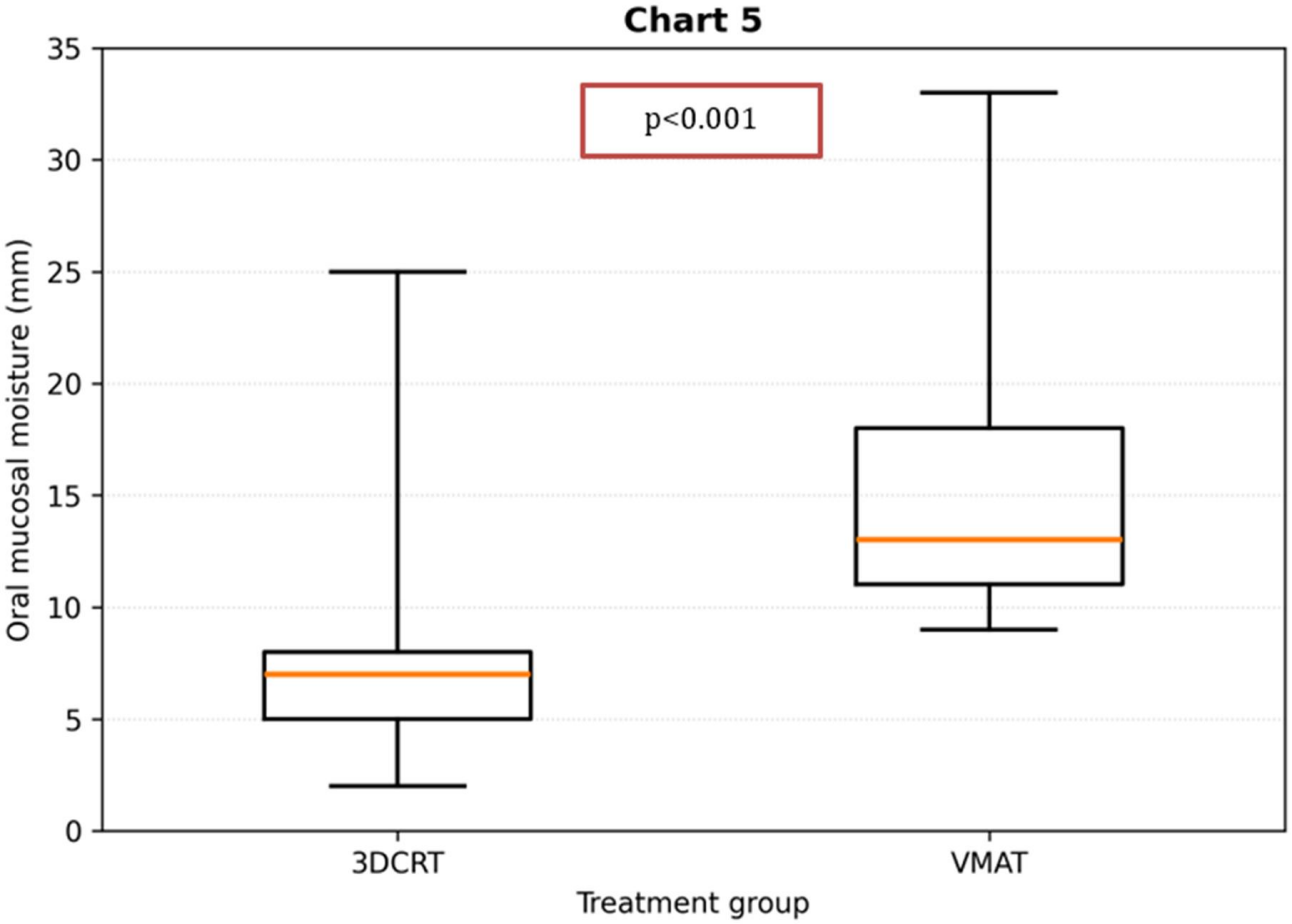
Analysis by groups (3DCRT/VMAT) and oral mucosal moisture.

## 4 Discussion

Salivary gland dysfunction is one of the most relevant complications of radiotherapy for head and neck cancer and may substantially affect oral health and daily functioning. Radiation-induced salivary dysfunction is influenced by several factors, including radiation dose, irradiated gland volume, tumor location, treatment technique, and individual patient characteristics. Modern intensity-modulated radiotherapy techniques, including VMAT, provide more conformal dose distributions and may facilitate greater sparing of functioning salivary gland tissue compared with conventional three-dimensional conformal radiotherapy (3DCRT) (5, 6).

In the present study, patients treated with VMAT demonstrated significantly higher unstimulated and stimulated whole salivary flow rates, stimulated parotid salivary flow, and oral mucosal moisture compared with patients treated with 3DCRT three months after completion of radiotherapy. These findings indicate higher objective salivary function in the VMAT cohort at the assessed post-treatment time point. The consistency of the findings across whole saliva, parotid secretion, and mucosal moisture suggests that the difference was not limited to a single salivary parameter.

The higher unstimulated salivary flow observed in the VMAT group is clinically relevant because resting saliva contributes continuously to oral lubrication and maintenance of mucosal integrity, whereas stimulated salivary flow reflects the functional secretory reserve of the salivary glands. The higher stimulated parotid secretion observed in the VMAT group further suggests greater residual parotid secretory activity after treatment. Previous studies have emphasized the importance of parotid gland dose in the development of post-radiotherapy salivary dysfunction (8, 9).

Radiation-induced salivary dysfunction may result from damage to acinar and ductal cells, vascular changes, and subsequent tissue remodeling. The clinical development and severity of post-radiotherapy xerostomia are multifactorial and may be influenced by patient-related, disease-related, and treatment-related factors (10). The extent of functional impairment is related to the dose received by functioning glandular tissue. In addition to the parotid glands, the submandibular and minor salivary glands may contribute to persistent salivary dysfunction. This is particularly relevant in patients with oropharyngeal malignancies, in whom treatment of the cervical nodal regions may result in radiation exposure to structures in the submandibular region (11). In particular, protection of the submandibular glands and minor salivary glands in the oral cavity has been associated with improved xerostomia-related outcomes in patients receiving intensity-modulated radiotherapy (9, 12).

The distinction between xerostomia and hyposalivation is important when interpreting these findings. Xerostomia refers to the subjective perception of oral dryness, whereas hyposalivation refers to objectively reduced salivary secretion. The present study assessed objective salivary gland function using unstimulated and stimulated whole salivary flow, stimulated parotid salivary flow, and oral mucosal moisture. Because no validated patient-reported xerostomia questionnaire was used, the present findings should not be interpreted as demonstrating a reduction in subjective xerostomia.

The findings of the present study are consistent with previous research demonstrating beneficial salivary and quality-of-life outcomes following parotid-sparing intensity-modulated radiotherapy. Lin et al. reported improved quality-of-life outcomes following parotid-sparing IMRT (13), while previous research has also demonstrated associations between salivary gland radiation dose and post-treatment salivary dysfunction (9, 12). In addition, our previous study reported differences in salivary flow and salivary lactoferrin following radiotherapy, supporting the importance of objective salivary assessment in patients treated for head and neck malignancies (14).

From a dosimetric perspective, VMAT differs from 3DCRT by allowing continuous modulation of radiation intensity during gantry rotation, potentially providing improved dose conformity and greater flexibility in sparing organs at risk while maintaining target coverage (6). The radiation dose received by tissues outside the target volume may also vary according to the radiation delivery technique and treatment configuration, emphasizing the importance of treatment planning and dose optimization for minimizing unnecessary radiation exposure to surrounding normal tissues (15). When the major salivary glands are appropriately delineated and included in treatment optimization, this increased conformality may reduce unnecessary radiation exposure to functioning glandular tissue. The observed higher salivary flow rates in the VMAT cohort may therefore be related, at least in part, to differences in the distribution of radiation dose to the salivary glands.

Nevertheless, these findings should be interpreted cautiously. The present study was cross-sectional, and salivary function was assessed only three months after radiotherapy. Pretreatment salivary measurements were not available, and therefore it cannot be determined whether the observed differences represent preservation of function relative to baseline or differences that were already present before treatment. Furthermore, detailed dosimetric parameters for the parotid and submandibular glands were not available for the present analysis. Differences in tumor characteristics, treatment volumes, surgical history, and other treatment-related factors may also influence salivary outcomes. Therefore, the present results demonstrate an association between treatment cohort and post-radiotherapy salivary function rather than definitive evidence of long-term glandular preservation.

The clinical relevance of these findings lies in the important role of saliva in maintaining oral homeostasis. Saliva contributes to lubrication and moistening of the oral mucosa, antimicrobial defense, buffering and remineralization of dental hard tissues, and facilitates speech, swallowing and mastication (1, 16). Persistent salivary hypofunction may consequently increase the risk of dental caries and periodontal disease and may contribute to taste disturbances, swallowing difficulties, impaired mastication, and nutritional problems (16–18). Thus, strategies that minimize unnecessary radiation exposure to functioning salivary tissue may have important implications for long-term oral health and supportive care in patients receiving head and neck radiotherapy. For patients who develop persistent radiation-induced xerostomia despite preventive treatment strategies, symptomatic management remains important. Horiot et al. reported relief of severe post-radiation xerostomia following treatment with pilocarpine, highlighting the potential role of pharmacological stimulation of residual salivary gland function (19).

Overall, the present study demonstrated significantly higher objective salivary flow rates and oral mucosal moisture in patients treated with VMAT compared with 3DCRT three months after radiotherapy. These findings support the potential benefit of modern intensity-modulated treatment in reducing the extent of post-treatment salivary dysfunction. However, prospective studies incorporating pretreatment and longitudinal salivary measurements, detailed salivary gland dosimetry, and validated patient-reported xerostomia and quality-of-life measures are required to further clarify the long-term clinical significance of these findings.

## 5 Conclusion

Modern radiotherapy techniques such as IMRT and VMAT offer improved dose conformity and the potential for greater sparing of salivary gland tissue during head and neck cancer treatment. In the present study, patients treated with VMAT demonstrated significantly higher unstimulated and stimulated whole salivary flow rates, stimulated parotid salivary flow, and oral mucosal moisture compared with patients treated with 3DCRT three months after radiotherapy. These findings suggest an association between VMAT treatment and better objective salivary function at the assessed post-treatment time point. However, given the cross-sectional study design and the absence of pretreatment salivary measurements and detailed salivary gland dosimetric data, these findings should not be interpreted as definitive evidence of long-term preservation or prevention of salivary dysfunction. Further prospective studies with longitudinal assessment, detailed dosimetric analysis, and validated patient-reported outcomes are warranted.

## Data Availability

The raw data supporting the conclusions of this article will be made available by the authors, without undue reservation.
